# Modulation of spinal motor networks by astrocyte-derived adenosine is dependent on D_1_-like dopamine receptor signaling

**DOI:** 10.1152/jn.00783.2017

**Published:** 2018-05-23

**Authors:** David Acton, Matthew J. Broadhead, Gareth B. Miles

**Affiliations:** School of Psychology and Neuroscience, University of St Andrews, St Andrews, United Kingdom

**Keywords:** central pattern generator, gliotransmission, metamodulation, motor control, neuromodulation, PAR1

## Abstract

Astrocytes modulate many neuronal networks, including spinal networks responsible for the generation of locomotor behavior. Astrocytic modulation of spinal motor circuits involves release of ATP from astrocytes, hydrolysis of ATP to adenosine, and subsequent activation of neuronal A_1_ adenosine receptors (A_1_Rs). The net effect of this pathway is a reduction in the frequency of locomotor-related activity. Recently, it was proposed that A_1_Rs modulate burst frequency by blocking the D_1_-like dopamine receptor (D_1_LR) signaling pathway; however, adenosine also modulates ventral horn circuits by dopamine-independent pathways. Here, we demonstrate that adenosine produced upon astrocytic stimulation modulates locomotor-related activity by counteracting the excitatory effects of D_1_LR signaling and does not act by previously described dopamine-independent pathways. In spinal cord preparations from postnatal mice, a D_1_LR agonist, SKF 38393, increased the frequency of locomotor-related bursting induced by 5-hydroxytryptamine and *N*-methyl-d-aspartate. Bath-applied adenosine reduced burst frequency only in the presence of SKF 38393, as did adenosine produced after activation of protease-activated receptor-1 to stimulate astrocytes. Furthermore, the A_1_R antagonist 8-cyclopentyl-1,3-dipropylxanthine enhanced burst frequency only in the presence of SKF 38393, indicating that endogenous adenosine produced by astrocytes during network activity also acts by modulating D_1_LR signaling. Finally, modulation of bursting by adenosine released upon stimulation of astrocytes was blocked by protein kinase inhibitor-(14–22) amide, a protein kinase A (PKA) inhibitor, consistent with A_1_R-mediated antagonism of the D_1_LR/adenylyl cyclase/PKA pathway. Together, these findings support a novel, astrocytic mechanism of metamodulation within the mammalian spinal cord, highlighting the complexity of the molecular interactions that specify motor output.

**NEW & NOTEWORTHY** Astrocytes within the spinal cord produce adenosine during ongoing locomotor-related activity or when experimentally stimulated. Here, we show that adenosine derived from astrocytes acts at A_1_ receptors to inhibit a pathway by which D_1_-like receptors enhance the frequency of locomotor-related bursting. These data support a novel form of metamodulation within the mammalian spinal cord, enhancing our understanding of neuron-astrocyte interactions and their importance in shaping network activity.

## INTRODUCTION

The cyclical activation of limb and trunk muscles during locomotion is directed by a central pattern generator (CPG) network within the ventral horn of the spinal cord. This network, like other CPGs, consists of interneurons that generate a stereotyped pattern of activity in motoneurons and hence muscles under a given set of conditions. By adjusting the conditions under which CPGs operate, including neuromodulator availability and excitation by descending inputs, animals are able to vary their motor behavior in response to the shifting demands imposed by their environment (Grillner 2003; Orlovsky et al. 1999). The mammalian locomotor CPG is modulated by a profusion of substances acting at multiple receptors, in this way vastly expanding its repertoire of possible outputs (Miles and Sillar 2011). This repertoire may be further expanded by interactions between neuromodulatory systems. Metamodulation, which has previously been described in the motor networks of *Xenopus* tadpoles (McLean and Sillar 2004) and lampreys (Song et al. 2012), is the modulation of a first-order modulator by a second-order modulator and may be important in refining the effects of a modulator that is widely released and has multiple targets (Katz 1999; Miles and Sillar 2011).

Adenosine, which in many systems derives from the hydrolysis of ATP, is a potent modulator of spinal motor networks (Acton and Miles 2017). Within isolated murine spinal cord (Acevedo et al. 2016; Acton and Miles 2015, 2017; Witts et al. 2012), adenosine derived from ATP acts at A_1_ adenosine receptors (A_1_Rs) to reduce the frequency of ongoing locomotor-related bursting, a mechanism first described in *Xenopus* tadpoles (Brown and Dale 2000; Dale and Gilday 1996). Neuromodulators may be released by neurons or astrocytes, which exhibit Ca^2+^-dependent release of so-called gliotransmitters (Araque et al. 1999, 2014; Bazargani and Attwell 2016). Astrocytes are proposed as the principal source of neuromodulatory adenosine in the ventral horn, since adenosinergic modulation does not occur when astrocytes are pharmacologically ablated (Acton and Miles 2015, 2017; Witts et al. 2012) and selective activation of the astrocytic receptor protease-activated receptor-1 (PAR1), a Gα_q_-linked G protein-coupled receptor, to stimulate Ca^2+^-dependent release of gliotransmitters, results in activation of A_1_Rs and modulation of neuronal activity (Acton and Miles 2015; Carlsen and Perrier 2014).

Adenosine modulates spinal motor circuitry in preparations lacking functionally relevant concentrations of dopamine (DA) (Acton and Miles 2017); however, it was recently proposed that A_1_Rs also modulate rhythmic network activity by inhibiting D_1_-like DA receptors (D_1_LRs) (Acevedo et al. 2016), an interaction previously observed in the basal ganglia (Popoli et al. 1996). A_1_Rs are tightly coupled to the Gα_i_ pathway, which mediates inhibition of adenylyl cyclase and reduces production of adenosine 3′,5′-cyclic monophosphate (cAMP). By contrast, D_1_LRs signal through Gα_s_ to stimulate adenylyl cyclase and cAMP production, resulting in activation of protein kinase A (PKA), which regulates diverse proteins, including ion channels and neurotransmitter receptors (Beaulieu and Gainetdinov 2011; Neve 2010). DA is known to be released within the ventral horn during locomotion (Gerin et al. 1995; Gerin and Privat 1998) and can modulate motor output by diverse mechanisms (Han and Whelan 2009; Humphreys and Whelan 2012; Madriaga et al. 2004; Sharples et al. 2014, 2015). Thus, DA may be an appropriate target for metamodulation within spinal motor circuitry.

In the spinal cord, A_1_ inhibition fails to alter the frequency of locomotor-related network activity when DA is absent or when D_1_LRs are inhibited (Acevedo et al. 2016). A_1_R blockade is similarly ineffective when PKA activity is inhibited (Acevedo et al. 2016), whereas modulation of locomotor-related bursting by A_1_Rs is restored when forskolin is applied to activate adenylyl cyclase independently of D_1_LR activation (Acevedo et al. 2016). Thus, adenosine is proposed to inhibit adenylyl cyclase in the spinal cord, counteracting signaling through the D_1_LR pathway to reduce the frequency of locomotor-related activity. However, D_1_LRs have not been shown to have the excitatory effect on network activity that is implied by this mechanism (Humphreys and Whelan 2012; Sharples et al. 2015). Furthermore, adenosine is reported to modulate neuronal intrinsic membrane properties and synaptic activity in the ventral horn in the absence of DA (Carlsen and Perrier 2014; Lloyd et al. 1988; Otsuguro et al. 2006, 2009, 2011; Taccola et al. 2012; Witts et al. 2012), and it has not been shown directly that adenosine derived from ATP released from astrocytes requires D_1_LR activation to modulate network activity.

Here, we assess potential interactions between astrocyte-derived adenosine and D_1_LR signaling during ongoing locomotor-related activity in isolated spinal cord preparations from postnatal mice. We first demonstrate that selective activation of D_1_LRs enhances the frequency of locomotor-related activity and that activation of D_1_LRs is necessary for the previously reported modulatory actions of bath-applied adenosine as well as endogenously produced adenosine acting at A_1_Rs. We then show that activation of PAR1 stimulates Ca^2+^ elevations in glial fibrillary acidic protein (GFAP)+ astrocytes and that adenosine produced after PAR1 activation modulates locomotor-related activity in a D_1_LR-dependent manner. Finally, we show that astrocyte-derived adenosine reduces burst frequency through a PKA-dependent mechanism, providing further support that it acts via the D_1_LR-Gα_s_ pathway.

## METHODS

### 

#### Tissue preparation.

All procedures performed on animals were conducted in accordance with the UK Animals (Scientific Procedures) Act 1986 and were approved by the University of St Andrews Animal Welfare and Ethics Committee. For electrophysiology recordings, spinal cords were isolated from postnatal day (P)1–4 C57BL/6 mice as previously described (Jiang et al. 1999). In summary, animals were killed by cervical dislocation, decapitated, and eviscerated before being transferred to a dissection chamber containing artificial cerebrospinal fluid (aCSF; equilibrated with 95% oxygen-5% carbon dioxide, ~4°C). Spinal cords were then isolated between midthoracic and upper sacral segments, and ventral and dorsal roots were trimmed. For Ca^2+^ imaging experiments, *Ai96*^LSL-GCaMP6s^ heterozygous mice (Jackson Laboratories; stock no. 024106) were crossed with *hGFAP::Cre-ERT2* hemizygous mice (*hGFAP::Cre*; Ganat et al. 2006) for glial imaging and *Pitx2^Cre^* heterozygous mice (Liu et al. 2003) for neuronal imaging. Cre expression in *hGFAP::Cre; Ai96*^LSL-GCaMP6s^ mice was induced by intraperitoneal injection of 4-hydroxytamoxifen (100 µg/g mouse wt) at P5 and P6, and P7 spinal cords were dissected as above. For slice preparation, lumbar spinal cord tissue was laid in 1% agar and a vibratome (Leica VT1200) was used to obtain 300-µm slices.

#### Ventral root recordings.

Isolated spinal cords were pinned ventral side up in a recording chamber perfused with aCSF (equilibrated with 95% oxygen-5% carbon dioxide; room temperature) at 10 ml/min. Glass suction electrodes were attached to the first or second lumbar ventral roots (L_1_, L_2_) on each side of the spinal cord to record flexor-related activity. Locomotor-related activity was evoked by bath application of 5-hydroxytryptamine (5-HT; 15 µM) and *N*-methyl-d-aspartate (NMDA; 5 µM) and was characterized by rhythmic bursting alternating between contralateral ventral roots. In some experiments, protein kinase inhibitor-(14–22)-amide (14–22 amide) or (±)-1-phenyl-2,3,4,5-tetrahydro-(1*H*)-3-benzazepine-7,8-diol hydrobromide (SKF 38393; 100 nM) was bath applied at the onset of locomotor-related bursting. All drugs present during the control period were also present during application of further drugs and during washout. In all experiments, stable rhythmic bursting was established over a period of ~1 h before the control period. Rhythmic bursting was considered stable when the frequency, amplitude, and duration of bursts were unchanged over several minutes. Data were amplified and filtered (band-pass filter 30–3,000 Hz; Qjin Design) and acquired at a sampling frequency of 6 kHz with a Digidata 1440A analog-to-digital converter and Axoscope software (Molecular Devices, Sunnyvale, CA). Custom-built amplifiers (Qjin Design) enabled simultaneous online rectification and integration (50 ms time constant) of raw signals.

#### Ca^2+^ imaging.

Ca^2+^ imaging was performed at ~34°C with a heated inflow of aCSF (equilibrated with 95% oxygen-5% carbon dioxide). Image acquisition was controlled with Micro-Manager 2 software. Images were acquired with a ×40 water immersion objective lens (0.9 numerical aperture) and a Zyla scientific CMOS camera (Andor, Oxford Instruments). Data were acquired with rolling shutter at either 1 or 2 frames/s with 150-ms exposure time. Illumination was provided by a 470-nm CoolLED system, which was controlled by the CMOS camera to deliver pulsed illumination during exposure times. Analysis of Ca^2+^ imaging was performed with ImageJ software. Sixteen-bit data were first converted to 8 bit and processed with a background subtraction and bleach correction with an open-source plugin. Active cells were selected and delineated manually from the images. Intensity measurements were converted to Δf/f_0_, calculated as 100 × (fluorescence value – baseline fluorescence ÷ baseline fluorescence). Baseline fluorescence was calculated as the mean average intensity from 10 to 20 frames before stimulation onset. Activity was quantified by measuring the average Δf/f_0_ for 4-min periods during control, drug [TFLLR-NH_2_ (TFLLR)], and wash phases.

#### Data analysis.

Data were analyzed off-line with DataView software (courtesy of Dr. W. J. Heitler, University of St Andrews). Ventral root bursts were identified from rectified/integrated traces, and their instantaneous frequencies, peak-to-peak amplitudes, and durations were then measured from the corresponding raw traces. Amplitude was measured as a noncalibrated unit and is presented here in arbitrary units. Statistical comparisons were performed on raw data averaged over 3-min periods for experiments testing the effects of PAR1 stimulation with TFLLR or 5-min periods in experiments to test other drugs. D’Agostino-Pearson tests were performed to assess normality. Normally distributed data were analyzed with repeated-measures ANOVA tests, unless otherwise indicated. Bonferroni post hoc tests were applied to pairwise comparisons. Where appropriate, sphericity was assessed with Mauchly’s test and Greenhouse-Giesser corrections were applied. Data sets lacking normal distribution were assessed by Friedman tests and are indicated in the text. For time course plots, data were averaged across 1-min bins and normalized to a 10-min precontrol period to permit comparison between preparations. Circular plots were used to assess the phase relationship between bursts recorded from the left and right sides of the spinal cord (Kjaerulff and Kiehn 1996) (statistiXL software, Nedlands, WA, Australia). For each preparation, 50 cycles were analyzed in control and drug conditions. Data points represent the mean phase of locomotor-related bursts recorded from right ventral roots. The beginning of the locomotor cycle is defined as the onset of left ventral root activity and has a value of 0. A value of 0.5 corresponds to strict alternation between right and left bursts. Rayleigh’s test for uniformity was used to assess mean burst onset time. Vector direction represents mean burst onset time, and vector length represents the concentration of data points around the mean. *P* values < 0.05 were considered significant. Tests were performed in SPSS Statistics for Windows, version 21.0 (IBM, Armonk, NY), GraphPad Prism 6 for Windows, version 6.01 (GraphPad Software, La Jolla, CA), or Excel 2013 (Microsoft, Redmond, WA).

#### Solution, drug, and enzyme preparation.

The aCSF used for dissections and recordings contained (in mM) 127 NaCl, 26 NaHCO_3_, 10 glucose, 3 KCl, 2 CaCl, 1.25 NaH_2_PO_4_, and 1 MgCl_2_. Adenosine, 4-hydroxytamoxifen, and TFLLR were supplied by Sigma-Aldrich (Poole, UK); 8-cyclopentyl-1,3-dipropylxanthine (DPCPX) was supplied by Abcam (Cambridge, UK); 14–22 amide, SKF 38393, and tetrodotoxin (TTX) were supplied by Tocris Bioscience (Bristol, UK). TFLLR, 14–22 amide, and SKF 38393 were dissolved in reverse-osmosis water; 4-hydroxytamoxifen was dissolved in corn oil; TTX was dissolved in citrate buffer; and adenosine and DPCPX were dissolved in DMSO. The concentration of DMSO in working solutions did not exceed 0.1% (vol/vol).

## RESULTS

### 

#### Selective activation of D_1_LRs increases frequency of locomotor-related network activity.

**Fig. 1.**
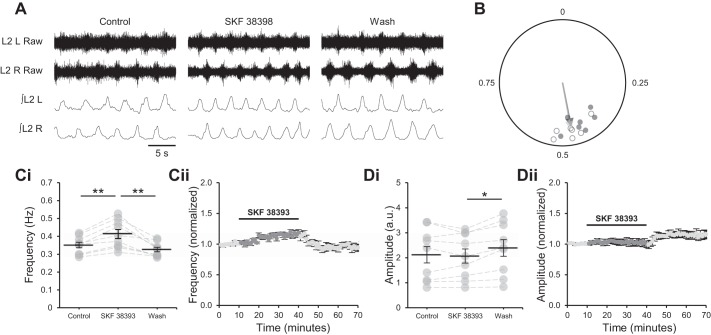
Activation of D_1_-like dopamine receptors (D_1_LRs) increases the frequency but not the amplitude of locomotor-related activity. *A*: raw (*top*) and rectified/integrated (*bottom*) traces recorded from left (L) and right (R) L_2_ ventral roots showing the effect of the selective agonist of D_1_LRs SKF 38393 (100 nM) on locomotor-related activity induced by 5-hydroxytryptamine (5-HT; 10 µM) and *N*-methyl-d-aspartate (NMDA; 5 µM). *B*: left-right phase relationship in control conditions and during application of SKF 38393. Data points represent the onset of locomotor bursts recorded from RL_2_ ventral roots in relation to the onset of activity recorded from corresponding LL_2_ roots (assigned a value of 0) in the same cycle. Circles represent mean values from individual experiments during control conditions (open) and during application of SKF 38393 (filled). Arrows represent means for all preparations (control, light gray; drug, dark gray). Vector direction indicates mean phase, and vector length corresponds to clustering of data points around the mean. *n* = 7 preparations. *Ci*: locomotor burst frequency over 5 min during a control period, during a 30-min application of SKF 38393, and during a 30-min washout. Individual data points are shown in gray, and means are represented by black lines. *n* = 9. *Cii*: time course plot of normalized data aggregated into 1-min bins showing an increase in burst frequency during SKF 38393 application. *n* = 9. *Di*: locomotor burst amplitude over 5 min during a control period, during a 30-min application of SKF 38393, and during a 30-min washout. *n* = 9. a.u., Arbitrary units. *Dii*: time course plot of normalized data aggregated into 1-min bins showing no change in burst amplitude during SKF 38393 application. *n* = 9. Error bars show ±SE. Statistically significant difference: *P < 0.05, **P < 0.01.

Adenosine is proposed to reduce the frequency but not the amplitude of locomotor-related activity by inhibiting signaling through the D_1_LR pathway (Acevedo et al. 2016), implying a role for D_1_LRs in the modulation of burst frequency but not amplitude. To determine the contribution of the D_1_LR pathway to the output of locomotor networks, the selective D_1_LR agonist SKF 38393 (100 nM) (Clemens et al. 2012; Neumeyer et al. 2003) was applied to isolated spinal cord preparations from P1–4 mice during stable locomotor-related activity induced by NMDA (5 µM) and 5-HT (10 µM). The frequency of bursts recorded from L_2_ ventral roots gradually increased over the 30-min period in which D_1_LRs were activated, with a maximum effect of 17.5 ± 2.6% (Fig. 1, *A* and *C*; *F*[2,16] = 18.9, *P* < 0.001, *n* = 9). The frequency returned to the baseline value when SKF 38393 was washed out (Fig. 1, *A* and *C*). Although burst amplitude was unchanged during application of SKF 38393, it increased after washout (15.1 ± 3.2%; Fig. 1, *A* and *D*; *F*[2,16] = 6.6, *P* < 0.01, *n* = 9). The phase relationship of alternating bursts in contralateral L_2_ roots was similar between control (Rayleigh’s test for uniformity: *P* < 0.001; *n* = 7) and drug (*P* < 0.001; *n* = 7) conditions (Fig. 1, *A* and *B*). These data indicate that D_1_LRs modulate the frequency of locomotor-related bursting but not its amplitude or bilateral phasing.

#### Activation of D_1_LRs is required for modulation of locomotor frequency by astrocyte-derived adenosine.

**Fig. 2.**
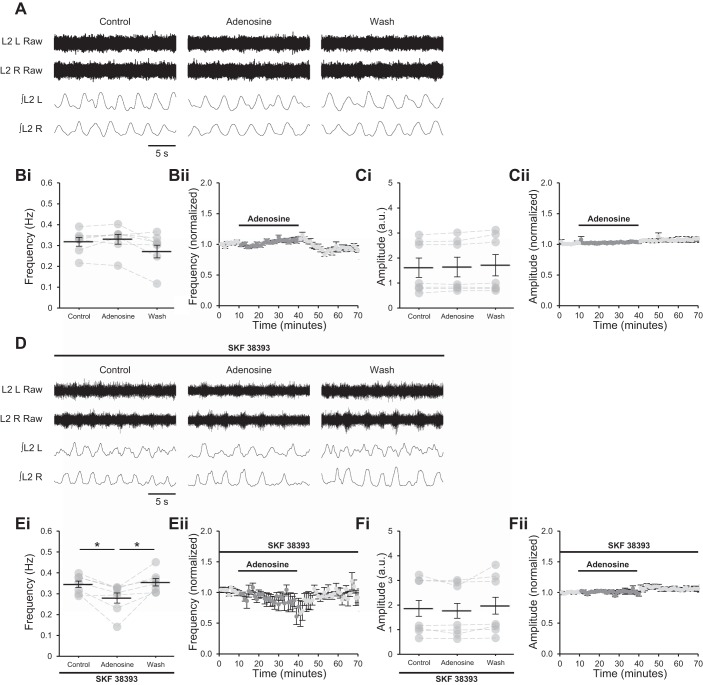
Adenosine requires activation of D_1_-like dopamine receptors (D_1_LRs) to modulate locomotor-related activity. *A*: raw (*top*) and rectified/integrated (*bottom*) traces recorded from left (L) and right (R) L_2_ ventral roots showing the effect of adenosine (75 µM) on locomotor-related activity induced by 5-hydroxytryptamine (5-HT; 10 µM) and *N*-methyl-d-aspartate (NMDA; 5 µM). *Bi*: locomotor burst frequency over 5 min during a control period, during a 30-min application of adenosine, and during a 30-min washout. Individual data points are shown in gray, and means are represented by black lines. *n* = 7 preparations. *Bii*: time course plot of normalized data aggregated into 1-min bins showing no change in burst frequency during adenosine application. *n* = 7. *Ci*: locomotor burst amplitude over 5 min during a control period, during a 30-min application of adenosine, and during a 30-min washout. *n* = 7. a.u., Arbitrary units. *Cii*: time course plot of normalized data aggregated into 1-min bins showing no change in burst amplitude during adenosine application. *n* = 7. *D*: raw (*top*) and rectified/integrated (*bottom*) traces recorded from LL_2_ and RL_2_ ventral roots showing the effect of adenosine on locomotor-related activity induced by 5-HT and NMDA in the presence of the selective agonist of D_1_LRs SKF 38393 (100 nM). *Ei*: locomotor burst frequency over 5 min during a control period, during a 30-min application of adenosine, and during a 30-min washout. SKF 38393 was present throughout. *n* = 7. *Eii*: time course plot of normalized data aggregated into 1-min bins showing a reduction in burst frequency during adenosine application in the presence of SKF 38393. *n* = 7. *Fi*: locomotor burst amplitude over 5 min during a control period, during a 30-min application of adenosine, and during a 30-min washout. SKF 38393 was present throughout. *n* = 7. *Fii*: time course plot of normalized data aggregated into 1-min bins showing no change in burst amplitude during adenosine application in the presence of SKF 38393. *n* = 7. Error bars show ±SE. Statistically significant difference: *P < 0.05.

**Fig. 3.**
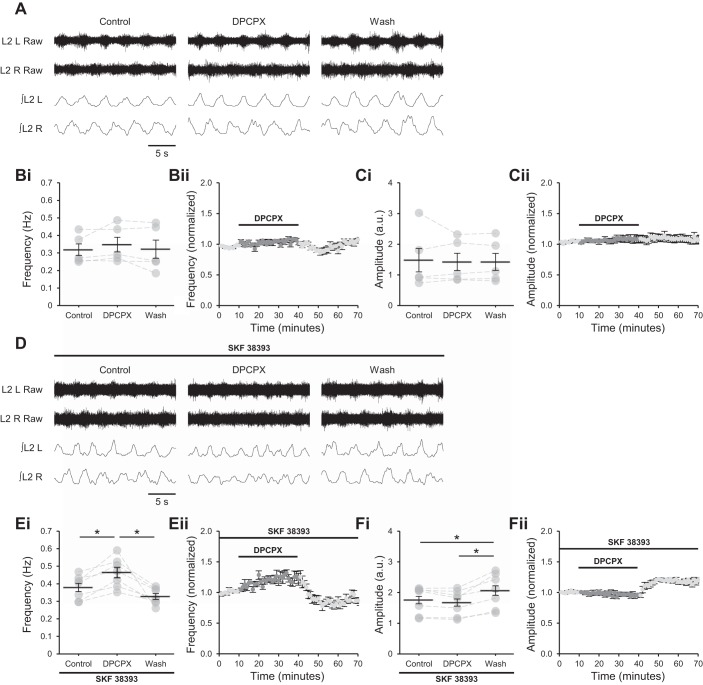
A_1_ adenosine receptors require activation of D_1_-like dopamine receptors (D_1_LRs) to modulate locomotor-related activity. *A*: raw (*top*) and rectified/integrated (*bottom*) traces recorded from left (L) and right (R) L_2_ ventral roots showing the effect of the selective A_1_ antagonist 8-cyclopentyl-1,3-dipropylxanthine (DPCPX; 1 µM) on locomotor-related activity induced by 5-hydroxytryptamine (5-HT; 10 µM) and *N*-methyl-d-aspartate (NMDA; 5 µM). *Bi*: locomotor burst frequency over 5 min during a control period, during a 30-min application of DPCPX (1–50 µM), and during a 30-min washout. Individual data points are shown in gray, and means are represented by black lines. *n* = 5 preparations. *Bii*: time course plot of normalized data aggregated into 1-min bins showing no change in burst frequency during DPCPX application. *n* = 5. *Ci*: locomotor burst amplitude over 5 min during a control period, during a 30-min application of DPCPX, and during a 30-min washout. *n* = 5. a.u., Arbitrary units. *Cii*: time course plot of normalized data aggregated into 1-min bins showing no change in burst amplitude during DPCPX application. *n* = 5. *D*: raw (*top*) and rectified/integrated (*bottom*) traces recorded from LL_2_ and RL_2_ ventral roots showing the effect of DPCPX (1 µM) on locomotor-related activity induced by 5-HT and NMDA in the presence of the selective agonist of D_1_LRs SKF 38393 (100 nM). *Ei*: locomotor burst frequency over 5 min during a control period, during a 30-min application of DPCPX (1 µM), and during a 30-min washout. SKF 38393 was present throughout. *n* = 7. *Eii*: time course plot of normalized data aggregated into 1-min bins showing an increase in burst frequency during DPCPX application in the presence of SKF 38393. *n* = 7. *Fi*: locomotor burst amplitude over 5 min during a control period, during a 30-min application of DPCPX, and during a 30-min washout. SKF 38393 was present throughout. *n* = 7. *Fii*: time course plot of normalized data aggregated into 1-min bins showing no change in burst amplitude during DPCPX application in the presence of SKF 38393. *n* = 7. Error bars show ±SE. Statistically significant difference: *P < 0.05.

Previous studies assessing the modulation of murine locomotor networks by adenosine were conducted in preparations in which DA was present (Acevedo et al. 2016; Acton and Miles 2015, 2017; Witts et al. 2012). However, blockade of D_1_LRs in the presence of DA was reported to prevent the modulation of burst frequency by adenosine (Acevedo et al. 2016). To confirm that activation of D_1_LRs is required for adenosinergic modulation in locomotor networks, adenosine (75 µM) (Witts et al. 2012) was bath applied to isolated spinal cords in which locomotor-related activity was induced by bath-applied NMDA and 5-HT. Importantly, descending dopaminergic neurons are severed in this preparation (Björklund and Skagerberg 1979; Commissiong et al. 1979; Hökfelt et al. 1979; Sharples et al. 2014; Skagerberg and Lindvall 1985), and DA is presumed to be absent, as DA receptor antagonists do not alter locomotor-related activity unless a DA receptor agonist is supplemented (Barrière et al. 2004). Adenosine modulated neither the frequency (Fig. 2, *A* and *B*; Friedman test: *P* > 0.05, *n* = 7) nor the amplitude (Fig. 2, *A* and *C*; *P* > 0.05, *n* = 7) of locomotor-related activity in these experiments. When adenosine was applied in the presence of SKF 38393, burst frequency was reduced (19.5 ± 6.0%; Fig. 2, *D* and *E*; *F*[2,12] = 10.7, *P* < 0.01, *n* = 7) but amplitude was unchanged (Fig. 2, *D* and *F*; *P* > 0.05, *n* = 7), as reported in preparations in which DA itself is present (Acevedo et al. 2016; Witts et al. 2012). Similarly, the A_1_R antagonist DPCPX (1–50 µM) (Acevedo et al. 2016; Witts et al. 2012), which blocks the actions of endogenously released adenosine to elicit an increase in burst frequency when DA is present (Acevedo et al. 2016; Witts et al. 2012), failed to modulate either the frequency (Fig. 3, *A* and *B*; *P* > 0.05, *n* = 7) or the amplitude (Fig. 3, *A* and *C*; *P* > 0.05, *n* = 7) of bursting in the absence of DA or SKF 38393. By contrast, DPCPX (1 µM) (Acevedo et al. 2016) applied in the presence of SKF 38398 elicited an increase in burst frequency (23.5 ± 4.8%; Fig. 3, *D* and *E*; *F*[2,12] = 13.7, *P* < 0.001, *n* = 7) without modulating burst amplitude; however, a modest increase in burst amplitude was recorded after washout of DPCPX (23.1 ± 2.9%; Fig. 3, *D* and *F*; *F*[2,12] = 17.9, *P* < 0.001, *n* = 7).

Selective activation of the endogenous astrocytic Gα_q_-linked G protein-coupled receptor PAR1 has been exploited to experimentally stimulate release of gliotransmitters throughout the central nervous system (Acton and Miles 2015, 2017; Carlsen and Perrier 2014; Lalo et al. 2014; Lee et al. 2007). Within the mouse spinal cord, where PAR1 receptors are selectively expressed by GFAP+ glia (Acton and Miles 2015), PAR1 activation triggers release of ATP, production of adenosine, and activation of A_1_Rs, which are reported to modulate the activity of ventral horn neurons in both the presence and the absence of DA (Acton and Miles 2015, 2017 Carlsen and Perrier 2014). We therefore next utilized activation of PAR1 by the selective agonist TFLLR to assess whether modulation of network activity by astrocyte-derived adenosine is dependent on D_1_LRs.

**Fig. 4.**
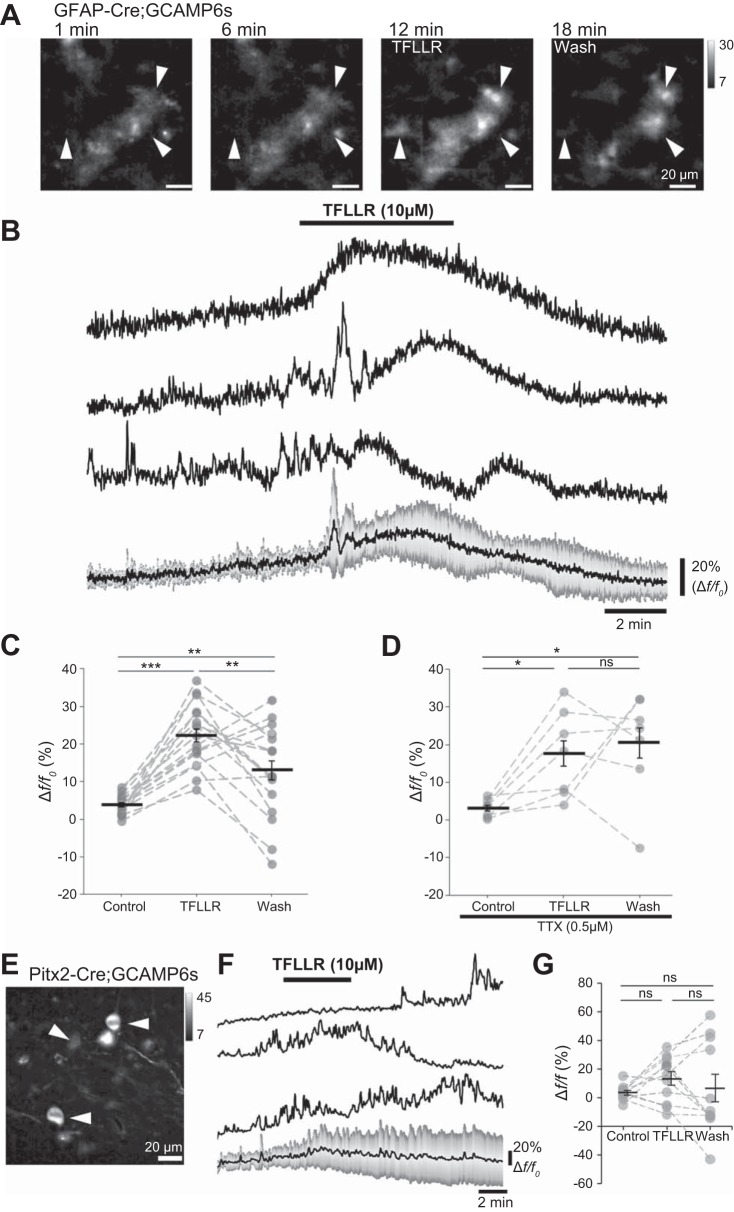
Protease-activated receptor-1 (PAR1) stimulation evokes Ca^2+^ signaling in spinal cord astrocytes. *A*: images of glial fibrillary acidic protein (GFAP)+ astrocytes expressing GCaMP6s in an acute spinal cord slice from a postnatal day (P)7 mouse (*hGFAP::Cre;GCAMP6s*), showing elevated intracellular Ca^2+^ after activation of PAR1 by TFLLR-NH_2_ (TFLLR; 10 µM) added between 7 and 12 min. Arrowheads denote 3 cells for which traces are shown in *B*. *B*: traces showing changes in Ca^2+^ levels in 3 astrocytes (*1–3*) during the addition of TFLLR to activate PAR1 receptors. Traces are plotted as Δf/f_0_. *Bottom*: mean Δf/f_0_ for 7 cells, with gray bars depicting the SD. *C*: average Δf/f_0_ during control, TFLLR, and wash periods plotted for cells pooled across 2 experiments performed on 2 different mice (*n* = 17 cells). *D*: average Δf/f_0_ during control, TFLLR, and wash periods plotted for cells imaged in slices incubated in tetrodotoxin (TTX; 0.5 µM; *n* = 7 cells across 2 experiments performed on 2 different mice). *E*: Pitx2^+^ interneurons expressing Cre-driven GCAMP6s. *F*: traces showing Ca^2+^ levels during application of TFLLR in 3 interneurons from a *Pitx2^Cre^;Ai96*^LSL-GCaMP6s^ animal and an averaged trace (*bottom*) from 11 cells (SD indicated in gray). *G*: average Δf/f_0_ values during control, TFLLR, and wash periods plotted for Pitx2^+^ interneurons from a *Pitx2^Cre^;Ai96*^LSL-GCaMP6s^ animal (data pooled from 4 slices). Error bars show ±SE. Statistically significant difference: *P < 0.05, **P < 0.01, ***P < 0.001. ns, Not significant.

Ca^2+^ imaging of GCaMP6s-expressing cells in acute lumbar spinal cord slices from P7 *hGFAP::Cre; Ai96*^LSL-GCaMP6s^ mice was first performed to further validate the use of PAR1 receptors to stimulate spinal GFAP+ astrocytes. Addition of TFLLR (10 µM; 5-min duration) induced prolonged (5–10 min) increases in astrocyte Ca^2+^ as indicated by positive changes in fluorescence intensity (Fig. 4, *A* and *B*). Robust Ca^2+^ responses were observed in spinal cord slices without TTX [Fig. 4*C*; *F*(2) = 19.1, *P* < 0.0001; *n* = 17 cells from 2 mice] and with 0.5 µM TTX [Fig. 4*D*; *F*(2) = 5.5, *P* < 0.05; *n* = 7 cells from 2 mice]. Furthermore, when visualizing the Ca^2+^ activity of a key interneuron subtype involved in locomotor modulation with *Pitx2^Cre^; Ai96*^LSL-GCaMP6s^ mice, it was observed there was no significant change in Ca^2+^ activity following TFLLR application [Fig. 4, *E*–*G*; *F*(2) = 0.63, *P* = 0.54; *n* = 11 cells]. These data suggest that TFLLR directly stimulates Ca^2+^ signaling in ventral horn astrocytes, acting independently of neuronal activity.

**Fig. 5.**
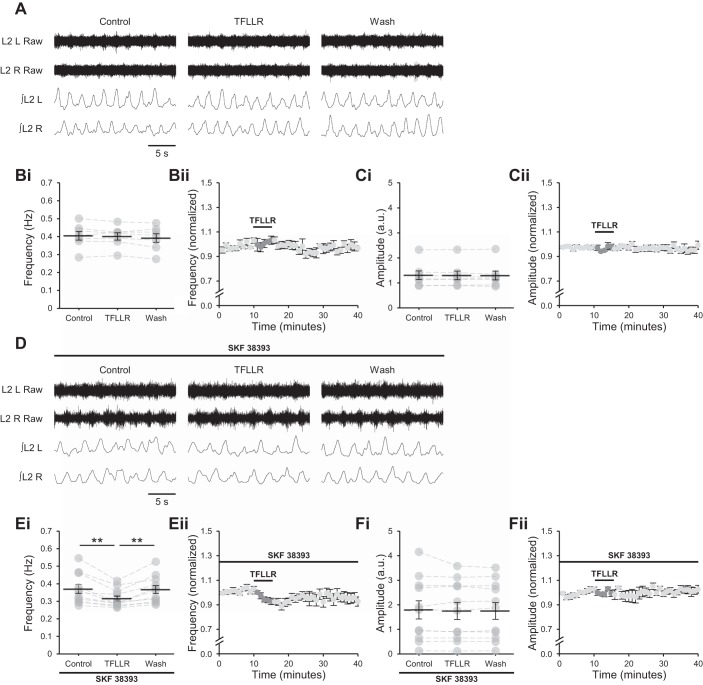
Adenosine released from astrocytes upon protease-activated receptor-1 (PAR1) activation requires activation of D_1_-like dopamine receptors (D_1_LRs) to modulate locomotor-related activity. *A*: raw (*top*) and rectified/integrated (*bottom*) traces recorded from left (L) and right (R) L_2_ ventral roots showing the effect of the PAR1 agonist TFLLR-NH_2_ (TFLLR; 10 µM) on locomotor-related activity induced by 5-hydroxytryptamine (5-HT; 10 µM) and *N*-methyl-d-aspartate (NMDA; 5 µM). *Bi*: locomotor burst frequency over 5 min during a control period, upon a 5-min application of TFLLR, and during a 25-min washout. Individual data points are shown in gray, and means are represented by black lines. *n* = 7 preparations. *Bii*: time course plot of normalized data aggregated into 1-min bins showing no change in burst frequency upon TFLLR application. *n* = 7. *Ci*: locomotor burst amplitude over 5 min during a control period, upon a 5-min application of TFLLR, and during a 25-min washout. *n* = 7. a.u., Arbitrary units. *Cii*: time course plot of normalized data aggregated into 1-min bins showing no change in burst amplitude upon TFLLR application. *n* = 7. *D*: raw (*top*) and rectified/integrated (*bottom*) traces recorded from LL_2_ and RL_2_ ventral roots showing the effect of TFLLR on locomotor-related activity induced by 5-HT and NMDA in the presence of the selective agonist of D_1_LRs SKF 38393 (100 nM). *Ei*: locomotor burst frequency over 5 min during a control period, upon a 5-min application of TFLLR, and during a 25-min washout. SKF 38393 was present throughout. *n* = 11. *Eii*: time course plot of normalized data aggregated into 1-min bins showing a transient reduction in burst frequency upon TFLLR application in the presence of SKF 38393. *n* = 11. *Fi*: locomotor burst amplitude over 5 min during a control period, upon a 5-min application of TFLLR, and during a 25-min washout. SKF 38393 was present throughout. *n* = 11. *Fii*: time course plot of normalized data aggregated into 1-min bins showing no change in burst amplitude upon TFLLR application in the presence of SKF 38393. *n* = 11. Error bars show ±SE. Statistically significant difference: **P < 0.01.

Bath application of TFLLR to stimulate astrocytes has been shown to reduce the frequency of ongoing locomotor-related activity in the presence of DA, without affecting burst amplitude or phase relationship, an effect attributed to enhanced release of astrocytic ATP and conversion of that ATP to adenosine (Acton and Miles 2015). In the present study, TFLLR (10 µM), like adenosine and DPCPX, failed to modulate the frequency (Fig. 5, *A* and *B*; *P* > 0.05, *n* = 7) or amplitude (Fig. 5, *A* and *C*; Friedman test: *P* > 0.05, *n* = 7) of locomotor-related activity in the absence of DA or SKF 38393. In the presence of SKF 38393, however, a transient reduction in burst frequency was detected (12.3 ± 2.3%; Fig. 5, *D* and *E*; *F*[2,20] = 14.9, *P* < 0.001, *n* = 11), with no change in amplitude (Fig. 5, *D* and *F*; *P* > 0.05, *n* = 11). Together, these results confirm that bath-applied adenosine, endogenous adenosine released in the spinal cord during locomotor-related activity, and adenosine released after stimulation of astrocytes modulate locomotor-related activity by a mechanism that requires the simultaneous activation of D_1_LRs.

#### Astrocyte-derived adenosine modulates locomotor-related activity in PKA-dependent manner.

**Fig. 6.**
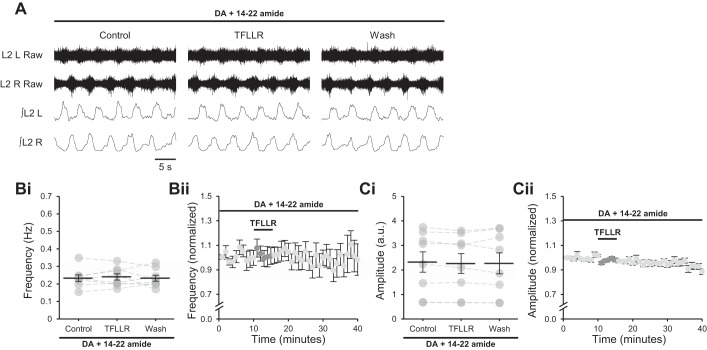
Adenosine released from astrocytes upon protease-activated receptor-1 (PAR1) activation requires protein kinase A (PKA) activity to modulate locomotor-related activity. *A*: raw (*top*) and rectified/integrated (*bottom*) traces recorded from left (L) and right (R) L_2_ ventral roots showing the effect of the PAR1 agonist TFLLR-NH_2_ (TFLLR; 10 µM) on locomotor-related activity induced by 5-hydroxytryptamine (5-HT; 10 µM), *N*-methyl-d-aspartate (NMDA; 5 µM) and dopamine (DA; 50 µM) in the presence of the PKA inhibitor protein kinase inhibitor-(14–22)-amide (14–22 amide; 1 µM). *Bi*: locomotor-burst frequency over 5 min during a control period, upon a 5-min application of TFLLR, and during a 25-min washout. Individual data points are shown in gray, and means are represented by black lines. *n* = 8 preparations. *Bii*: time course plot of normalized data aggregated into 1-min bins showing no change in burst frequency upon TFLLR application. *n* = 8. *Ci*: locomotor burst amplitude over 5 min during a control period, upon a 5-min application of TFLLR, and during a 25-min washout. *n* = 8. a.u., Arbitrary units. *Cii*: time course plot of normalized data aggregated into 1-min bins showing no change in burst amplitude upon TFLLR application. *n* = 8. Error bars show ±SE.

D_1_LRs signal through Gα_s_ to stimulate adenylyl cyclase and cAMP production, resulting in activation of PKA and the modulation of neuronal activity (Beaulieu and Gainetdinov 2011; Neve 2010), whereas A_1_Rs signal through Gα_i_ and are proposed to inhibit adenylyl cyclase and its downstream effectors, acting in opposition to D_1_LRs (Acevedo et al. 2016). This suggests that adenosine released after astrocytic stimulation modulates network activity by reducing the activity of PKA. To confirm this, TFLLR was applied to spinal cord preparations in which locomotor-related activity had been induced by NMDA (5 µM), 5-HT (10 µM), and DA (50 µM) in the presence of 14–22 amide (1 µM), a PKA inhibitor (Acevedo et al. 2016). Under these conditions, the modulation of burst frequency upon PAR1 activation was abolished (Fig. 6, *A* and *B*; *P* > 0.05, *n* = 8), and burst amplitude was also unchanged (Fig. 6, *A* and *C*; *P* > 0.05, *n* = 8). These data corroborate the inhibition of signaling through the D_1_LR/adenylyl cyclase/PKA pathway by astrocyte-derived adenosine.

## DISCUSSION

The experiments described in this study indicate that endogenous adenosine derived from astrocytes likely functions during locomotor-related activity as a second-order modulator, constraining excitatory DA signaling mediated through D_1_LRs and PKA. Furthermore, we found no evidence that astrocyte-derived adenosine acts during ongoing locomotor activity via the DA-independent mechanism or mechanisms previously detected in spinal cord preparations (Carlsen and Perrier 2014; Lloyd et al. 1988; Otsuguro et al. 2006, 2009, 2011; Taccola et al. 2012; Witts et al. 2012). Metamodulation of this kind may serve to provide acute control over a modulator that has very broad effects within the spinal cord (Katz 1999; Sharples et al. 2014).

Adenosine acting at A_1_Rs is proposed to act in opposition to DA acting at D_1_LRs to reduce the speed of locomotion. This implies a previously undemonstrated excitatory role for D_1_LRs in modulating the frequency of locomotor-related activity in mice. Accordingly, selective activation of D_1_LRs with SKF 38393 is shown here to increase the frequency of locomotor-related activity. This finding contradicts previous studies reporting no change in the frequency of locomotor-related activity when D_1_LRs are activated by another phenyl-benzazepine A_1_ agonist, SKF 81297 (Humphreys and Whelan 2012; Sharples et al. 2015). However, SKF 81297 applied at the concentration used in these studies is also likely to activate inhibitory D_2_LRs (Neumeyer et al. 2003), which have been shown to reduce the frequency of locomotor-related bursting (Humphreys and Whelan 2012; Sharples et al. 2015). The excitatory effect of D_1_LRs on burst frequency may therefore have been masked in these studies. In addition, frequency modulation by D_1_LRs was recorded in the present study after 25 min and had a relatively slow onset; it may therefore have been missed by previous studies using shorter application periods. The evidence presented here that D_1_LRs are indeed excitatory in postnatal mice is consistent with studies showing that D_1_LRs stimulate locomotor activity in intact adult mice (Lapointe et al. 2009) and neonatal rats (Barrière et al. 2004). Conversely, it is shown that bath-applied adenosine, which acts via A_1_ but not A_2A_ receptors in the murine locomotor circuitry (Acevedo et al. 2016; Acton and Miles 2015; Carlsen and Perrier 2014; Witts et al. 2012), reduces the frequency of locomotor-related activity, whereas A_1_ blockade increases it. Importantly, the effects of A_1_R activation or inhibition are only observed when D_1_LRs are also activated.

D_1_LR activation does not alter the phase relationship between bursts in contralateral roots, as previously reported for adenosine (Witts et al. 2012). In addition, despite evidence that D_1_LRs enhance AMPA currents in motoneurons (Han and Whelan 2009), no change in the amplitude of locomotor-related bursting is detected upon activation of either D_1_LRs or A_1_Rs (Acevedo et al. 2016; Acton and Miles 2015; Carlsen and Perrier 2014; Witts et al. 2012). However, although burst amplitude is unaltered during activation of D_1_LRs and during inhibition of A_1_Rs by DPCPX, in both cases burst amplitude increases upon drug washout. The mechanism by which this occurs is unclear, but it may be relevant that in both experiments the change in amplitude follows a period of enhanced signaling through the D_1_LR pathway; in the case of DPCPX, this is likely to occur because of A_1_ blockade relieving inhibition of the D_1_LR pathway by endogenous adenosine. In summary, the data presented here indicate that A_1_Rs and D_1_LRs have opposite effects on the frequency of locomotor-network activity and that neither receptor modulates the amplitude or left-right phasing of locomotor-related output. This is consistent with the proposal that they act via a common pathway (Acevedo et al. 2016).

Previous studies demonstrated that A_1_Rs modulate the frequency of ongoing network activity in murine preparations in the presence of DA (Acevedo et al. 2016; Acton and Miles 2015; Carlsen and Perrier 2014; Witts et al. 2012) but not in the presence of a D_1_LR inhibitor (Acevedo et al. 2016). Consistent with these reports, blockade of A_1_Rs fails to modulate activity in the rat spinal cord in the absence of DA (Taccola et al. 2012). In the present study, it is shown that the previously reported effects of adenosine, DPCPX, and PAR1 activation by TFLLR on network activity are absent when DA is excluded from the aCSF but are restored in the presence of a D_1_LR agonist. Thus, activation of D_1_LRs is a precondition for adenosinergic modulation of ongoing locomotor-related activity. However, it is not certain how these findings relate to previous observations that adenosine modulates postsynaptic currents in interneurons in acute slices from postnatal mice, both when bath applied at the same concentration as used here (Witts et al. 2015) and when released endogenously after stimulation of astrocytes (Carlsen and Perrier 2014). Furthermore, adenosine applied to isolated rat spinal cords in the absence of DA modulates burst amplitude during locomotor-related activity, the frequency of disinhibited bursting, and the duration of bouts of locomotor-related activity induced by dorsal root stimulation (Taccola et al. 2012). Adenosine also modulates reflex potentials induced by dorsal root stimulation in rats (Lloyd et al. 1988; Otsuguro et al. 2006, 2009, 2011; Taccola et al. 2012). All of these DA-independent effects are abolished by A_1_ blockade; however, in these studies A_1_ blockade alone has no effect in the absence of DA, indicating that adenosine present at basal levels within spinal cord preparations does not have modulatory effects. It is therefore possible that a high concentration of exogenous adenosine can under some circumstances act independently of DA. Alternatively, however, the DA-independent effects of adenosine recorded at the cellular level in slices from postnatal mice (Witts et al. 2012) might be too weak to affect whole-network output unless amplified by coactivation of D_1_LRs, or they might simply be irrelevant to the production of locomotor-related activity. It is also possible that a state-dependent mechanism is involved, such that the effects of adenosine on synaptic transmission and resting membrane potential that are observed in slices are not produced during network activity. Further experiments in which the cellular consequences of manipulation of D_1_LR and A_1_R signaling are investigated in slice preparations may be able to resolve this discrepancy.

Microdialysis experiments have shown that DA is released in the spinal cord during locomotion (Gerin et al. 1995; Gerin and Privat 1998). In addition, adenosinergic modulation scales with neuronal activity (Acton and Miles 2015), which may reflect activity-dependent release. One possibility is that the excitatory actions of DA and the inhibitory actions of adenosine do not perfectly overlap, such that DA promotes locomotion at low frequencies of network activity but its effect is constrained by adenosine at higher frequencies of network activity. Adenosine may function in this way to stabilize network output, ensuring controlled locomotion at higher speeds, or to prevent metabolic exhaustion. Alternatively, adenosinergic inhibition of D_1_LR signaling may be balanced with proportionate dopaminergic activation across all speeds of locomotion, such that adenosine acts to shape dopaminergic modulation by limiting only some of its actions. In both scenarios, second-order adenosinergic modulation may represent an efficient and selective mechanism of control over a potent first-order modulator that has diverse actions within a network (Katz 1999). In the case of DA, those actions are mediated by multiple cell types via both D_1_LRs and D_2_LRs. It is possible that adenosine release is highly localized and that it modulates only a subset of D_1_LRs. Consistent with this, it was previously observed that motoneurons and excitatory interneurons are insensitive to adenosine during synchronous network activity produced by blockade of inhibitory transmission, implying that adenosine acts via inhibitory interneurons to modulate burst frequency (Acton and Miles 2015; Witts et al. 2012). This might suggest that adenosine modulates only signaling through D_1_LRs expressed by inhibitory interneurons; however, further experiments are required to conclusively identify the cellular targets of both DA and adenosine during locomotion.

Inhibition of PKA prevents the modulation of locomotor-related bursting otherwise detected after stimulation of astrocytes. Similarly, PKA inhibition abolishes the effect of A_1_ blockade (Acevedo et al. 2016). These findings support a role for PKA in the modulation of network activity by adenosine acting through the D_1_LR pathway. PKA has diverse molecular targets, including ion channels and neurotransmitter receptors (Beaulieu and Gainetdinov 2011; Neve 2010). Although AMPA receptors may be modulated by PKA (Banke et al. 2000; Esteban et al. 2003) and motoneuronal AMPA receptors are modulated by D_1_LRs in the lumbar spinal cord (Han and Whelan 2009), motoneuronal AMPA receptors are unlikely to mediate the effects of adenosine during locomotor-related activity (see above). The targets of PKA activity, which may include AMPA receptors expressed by inhibitory interneurons, therefore remain to be elucidated.

The data presented here provide evidence that adenosine produced upon stimulation of astrocytes acts via A_1_Rs to inhibit signaling through D_1_LRs. Significantly, astrocytes are proposed as the principal source of modulatory adenosine in spinal motor networks (Acton and Miles 2015, 2017; Witts et al. 2012). These findings support a previously described interaction between A_1_ and D_1_LRs in spinal motor networks (Acevedo et al. 2016). Other examples of metamodulation in spinal locomotor networks are provided by *Xenopus* tadpoles, in which nitric oxide modulates the release of norepinephrine (McLean and Sillar 2004), and lampreys, in which nitric oxide modulates the activity of endocannabinoids (Song et al. 2012). The present study illuminates the complexity of neuron-astrocyte cross talk in spinal motor networks and demonstrates a mechanism by which a second-order neuromodulator refines the effects of a first-order neuromodulator with diverse and potent actions, providing behaviorally relevant network output with flexibility and specificity.

## GRANTS

D. Acton was supported by funds from a Wellcome Trust Institutional Strategic Support Fund grant. G. B. Miles and M. J. Broadhead received support from Biotechnology and Biological Science Research Grant BB/M021793/1.

## DISCLOSURES

No conflicts of interest, financial or otherwise, are declared by the authors.

## AUTHOR CONTRIBUTIONS

D.A. and G.B.M. conceived and designed research; D.A. and M.J.B. performed experiments; D.A. analyzed data; D.A., M.J.B. and G.B.M. interpreted results of experiments; D.A. and M.J.B prepared figures; D.A. and M.J.B. drafted manuscript; D.A., M.J.B. and G.B.M. edited and revised manuscript; D.A., M.J.B. and G.B.M. approved final version of manuscript.
